# Valvular Heart Disease and Heart Failure in the Post-COVID-19 Era: A Narrative Review of Mechanisms, Diagnosis, Differential Assessment, and Clinical Outcomes

**DOI:** 10.3390/jcm15135007

**Published:** 2026-06-26

**Authors:** Maria Rada, Iasmina Madalina Petculescu, Ana-Maria Pah, Adina Avram, Dana Emilia Velimirovici, Ariana Bianca Velciov, Cristina Tudoran, Stela Iurciuc, Diana Utu, Dan Radu Gheorghe, Maria-Laura Craciun

**Affiliations:** 1 Cardiology Department, “Victor Babes” University of Medicine and Pharmacy, Eftimie Murgu Square 2, 300041 Timisoara, Romania; rada.maria@umft.ro (M.R.); dana.velimirovici@umft.ro (D.E.V.); iurciuc.stela@umft.ro (S.I.); laura.craciun@umft.ro (M.-L.C.); 2 Department of Materials and Fabrication Engineering, Politehnica University Timisoara, Boulevard Mihai Viteazu, Number 1, 300222 Timisoara, Romania; iasmina.petculescu@upt.ro; 3 Department of Internal Medicine I, “Victor Babes” University of Medicine and Pharmacy, Eftimie Murgu, Square 2, 300041 Timisoara, Romania; 4 Food Science Department, University of Life Sciences “King Michael I” Timisoara, Calea Aradului, 300645 Timisoara, Romania; arianavelciov@usvt.ro; 5 Department VII, Internal Medicine II, “Victor Babes” University of Medicine and Pharmacy, Eftimie Murgu Square, Number 2, 300041 Timisoara, Romania; tudoran.cristina@umft.ro; 6 Department II, Physiology and Pathophysiology, “Victor Babes” University of Medicine and Pharmacy, Eftimie Murgu Square 2, 300041 Timisoara, Romania; diana.utu@umft.ro; 7 Department of Surgery I, “Victor Babes” University of Medicine and Pharmacy, Eftimie Murgu Square 2, 300041 Timisoara, Romania; radu.dan@umft.ro

**Keywords:** COVID-19, valvular heart disease, heart failure, functional mitral regurgitation, endothelial dysfunction, NT-proBNP, post-acute sequelae of SARS-CoV-2, HFpEF, echocardiography

## Abstract

**Background/Objectives**: Cardiovascular involvement is among the most consequential sequelae of SARS-CoV-2 infection. Myocardial injury, arrhythmia, and thromboembolic disease have been characterized in depth, yet the relationship between COVID-19 and valvular heart disease (VHD), and its interplay with heart failure (HF), has received comparatively limited synthesis. This narrative review consolidates current evidence on the mechanisms, diagnosis, differential assessment, and clinical outcomes linking acute and post-acute COVID-19 to valvular dysfunction and to incident or worsening heart failure, with emphasis on practical implications for cardiologists and internists. **Methods**: We searched PubMed, Scopus, and Web of Science (January 2020–January 2026) for studies on valvular dysfunction, heart failure, myocardial injury, and endothelial pathology in SARS-CoV-2 infection, and synthesized findings narratively. **Results**: Convergent pathways—endothelial injury, systemic hyperinflammation, micro- and macrovascular thrombosis, and pressure–volume overload—contribute to functional and, less frequently, structural valvular changes. Available evidence suggests that clinically relevant post-COVID valvular abnormalities are more often secondary/functional (mitral and tricuspid regurgitation) than primary structural lesions, although dedicated prospective valvular studies remain scarce. Pre-existing severe VHD markedly worsens acute COVID-19 prognosis. Elevated NT-proBNP, troponin, and interleukin-6 consistently predict decompensation and mortality, and a substantial minority of survivors show persistent fibrotic pulmonary changes and restrictive ventilatory defects on follow-up (pulmonary rather than cardiac findings). **Conclusions**: Post-COVID valvular dysfunction appears, on currently available but largely indirect evidence, predominantly functional and inflammation-related, and may overlap with HFpEF phenotypes in selected patients when objective diagnostic criteria are fulfilled. Biomarker-guided, multimodality follow-up is reasonable in high-risk survivors, and prospective longitudinal studies with standardized valvular endpoints remain a priority. Dedicated longitudinal evidence on valvular outcomes specifically remains very limited.

## 1. Introduction

Severe acute respiratory syndrome coronavirus 2 (SARS-CoV-2) imposes a cardiovascular burden that extends well beyond the acute respiratory phase. The virus engages the cardiovascular system through angiotensin-converting enzyme 2 (ACE2), expressed on cardiomyocytes, endothelial cells, and valvular interstitial cells [1,2]. Myocardial injury—defined by troponin elevation—was reported in at least 10% of hospitalized patients and up to 41% of critically ill patients or those with cardiovascular comorbidity. This injury consistently conferred excess mortality [3,4]. As the acute crisis matured into a recognition of post-acute sequelae of SARS-CoV-2 infection (PASC), attention shifted toward durable cardiac consequences experienced by survivors [5,6].

The link between COVID-19 and heart failure (HF) is now well supported: a large national cohort described elevated 12-month hazards for incident HF among survivors relative to controls, even in those not hospitalized acutely [5]. By contrast, the relationship between SARS-CoV-2 and valvular heart disease (VHD) has received markedly less attention. This is an important gap, because the mechanisms implicated in COVID-19 cardiac injury—endothelial dysfunction, microthrombosis, and intense systemic inflammation—overlap substantially with those that contribute to functional valvular regurgitation and to the transition to decompensated heart failure, particularly heart failure with preserved ejection fraction (HFpEF) [7,8,9].

Two clinically distinct scenarios must be separated. The first is the effect of pre-existing VHD on acute COVID-19 outcome; an international registry reported 30-day mortality approaching 50% in patients hospitalized with concomitant severe VHD and COVID-19 [10]. The second, less well characterized, is the emergence or worsening of functional valvular regurgitation as a consequence of COVID-19 itself—arising from ventricular and annular distortion, pulmonary hypertension, and right-heart pressure overload rather than from intrinsic leaflet disease [11,12]. Distinguishing incidental degenerative VHD from post-COVID functional regurgitation is a recurrent and consequential clinical problem, especially in elderly patients in whom degenerative disease is prevalent.

This narrative review is organized around four clinical questions: (i) the influence of pre-existing VHD on the prognosis of acute COVID-19; (ii) acute, often transient and potentially reversible hemodynamic valvular dysfunction during the acute illness; (iii) persistent post-acute myocardial or valvular abnormalities; and (iv) incident heart failure after recovery; diagnostic imaging and the differentiation of incidental degenerative VHD are addressed thereafter. Throughout, we contextualize international evidence with regional cohort data from a tertiary center in Eastern Europe, where pulmonary embolism, acute pulmonary edema, and sepsis biomarkers have been systematically characterized in hospitalized COVID-19 populations [13,14,15,16].

## 2. Materials and Methods

### 2.1. Search Strategy and Information Sources

This is a narrative review. We searched PubMed/MEDLINE, Scopus, and Web of Science for English-language articles published between 1 January 2020 and 31 January 2026. The search combined controlled vocabulary and free-text terms across two domains: the exposure (“COVID-19”, “SARS-CoV-2”, “post-acute COVID-19”, “long COVID”, “PASC”) and the cardiac outcome (“valvular heart disease”, “mitral regurgitation”, “tricuspid regurgitation”, “aortic stenosis”, “heart failure”, “HFpEF”, “myocardial injury”, “endothelial dysfunction”, “NT-proBNP”, “echocardiography”, “cardiac magnetic resonance”). Reference lists of retrieved articles and prior reviews were screened manually. Terms within each domain were combined with the Boolean operator OR, and the two domains were combined with AND; the indicative string applied in PubMed/MEDLINE was (“COVID-19” OR “SARS-CoV-2” OR “post-acute COVID-19” OR “long COVID” OR “PASC”) AND (“valvular heart disease” OR “mitral regurgitation” OR “tricuspid regurgitation” OR “aortic stenosis” OR “heart failure” OR “HFpEF” OR “myocardial injury” OR “endothelial dysfunction” OR “NT-proBNP” OR “echocardiography” OR “cardiac magnetic resonance”), with analogous syntax adapted to each database. In Scopus the string was TITLE-ABS-KEY((“COVID-19” OR “SARS-CoV-2” OR “post-acute COVID-19” OR “long COVID” OR “PASC”) AND (“valvular heart disease” OR “mitral regurgitation” OR “tricuspid regurgitation” OR “aortic stenosis” OR “heart failure” OR “HFpEF” OR “myocardial injury” OR “endothelial dysfunction” OR “NT-proBNP” OR “echocardiography” OR “cardiac magnetic resonance”)), and in Web of Science the Topic string was TS = ((“COVID-19” OR “SARS-CoV-2” OR “post-acute COVID-19” OR “long COVID” OR “PASC”) AND (“valvular heart disease” OR “mitral regurgitation” OR “tricuspid regurgitation” OR “aortic stenosis” OR “heart failure” OR “HFpEF” OR “myocardial injury” OR “endothelial dysfunction” OR “NT-proBNP” OR “echocardiography” OR “cardiac magnetic resonance”)). The final search was executed on 31 January 2026. Records retrieved across the three databases were de-duplicated manually on the basis of title, author list, and DOI. Titles and abstracts, and subsequently full texts, were screened independently by two reviewers (M.R. and A.-M.P.); disagreements were resolved by discussion until consensus was reached. Approximately 41 articles were ultimately retained for qualitative synthesis. Consistent with the narrative design, studies were prioritized pragmatically according to direct relevance to valvular or heart-failure outcomes, methodological quality, recency, and contemporary guideline status, rather than by a formal quantitative weighting scheme. The conduct and reporting of this review were guided by the Scale for the Assessment of Narrative Review Articles (SANRA), and a structured map of the evidence underpinning the synthesis, classified by directness and strength, is provided in Table 1.

### 2.2. Eligibility and Selection

We included observational cohorts, case–control studies, mechanistic and pathological studies, imaging studies, registries, systematic reviews, and authoritative clinical guidelines addressing valvular dysfunction, heart failure, or the relevant cardiac mechanisms in SARS-CoV-2 infection. Case reports were included only to illustrate mechanisms not otherwise documented. Preprints were excluded unless subsequently peer-reviewed. Because the review is narrative rather than systematic, no quantitative pooling was performed, and no PROSPERO registration, formal PICO framework, or meta-analytic risk-of-bias scoring was applied. This design was chosen deliberately given the mechanistic breadth and heterogeneity of the literature.

### 2.3. Data Synthesis

Evidence was organized thematically into mechanism, diagnosis, differential assessment, and outcomes, followed by a dedicated discussion of clinical implications. Where regional data were available, international findings were contextualized with cohort results from hospitalized COVID-19 populations in Romania [13,14,15,16]. These regional cohorts are used throughout as illustrative, single-center examples that lend quantitative texture—prevalence, biomarker thresholds, and follow-up imaging—to mechanisms otherwise discussed qualitatively, and not as definitive or representative estimates of the broader international post-COVID valvular disease population. Conflicting findings are presented explicitly, and the strength of underlying evidence is qualified throughout, with deliberately cautious wording where direct valvular data are sparse.

## 3. Results

To separate distinct clinical questions that are frequently conflated, the evidence below is organized around four questions: (i) the influence of pre-existing valvular heart disease on the prognosis of acute COVID-19; (ii) acute, often transient and potentially reversible hemodynamic valvular dysfunction arising during the acute illness; (iii) persistent post-acute myocardial or valvular abnormalities; and (iv) incident heart failure after recovery. For the purpose of this review, we adopt the NICE temporal framework, which distinguishes ongoing symptomatic COVID-19 (signs or symptoms from 4 to 12 weeks after onset) from post-COVID-19 syndrome (signs or symptoms that develop during or after COVID-19, continue for more than 12 weeks, and are not explained by an alternative diagnosis). This differs from the World Health Organization post-COVID-19 condition definition, under which symptoms usually arise within three months of the initial infection and persist for at least two months; the two definitions are therefore presented separately rather than merged. The four subsections that follow address questions (i) through (iv) in turn, with diagnostic imaging and differential assessment treated separately thereafter, and the directness and strength of the evidence supporting each are summarized in Table 1. Because few of the available studies were designed with valvular endpoints, the proposition that functional mitral and tricuspid regurgitation represent the predominant post-COVID valvular phenotype is presented as hypothesis-generating rather than as established fact.

### 3.1. Pre-Existing Valvular Heart Disease and Acute COVID-19 Prognosis

Patients with known significant VHD warrant particular vigilance, given the markedly worse acute outcomes observed in registry data [10,21]. During acute infection, the priorities are careful volume and rate management, recognition that fixed obstructive lesions such as severe aortic stenosis tolerate tachycardia and hypovolemia poorly, and early multidisciplinary (heart-team) discussion when decompensation occurs. The registry experience suggests that, in selected patients with severe aortic stenosis, valve intervention performed early after infection was associated with lower 30-day mortality than conservative management, although the small, uncontrolled nature of these data precludes firm causal conclusions [10]. After recovery, management reverts to standard valvular guideline pathways, with the added consideration that any apparent worsening of regurgitation should be re-evaluated once the acute inflammatory and high-output state has resolved [22].

Secondary mitral regurgitation, one of the central phenotypes of this review, deserves separate emphasis because the pandemic created a particularly difficult situation for these patients: delayed evaluation, reduced access to elective procedures, and frailty or pulmonary comorbidity could all worsen outcomes. Since post-COVID mitral regurgitation is predominantly functional, its assessment should be performed after recovery rather than during the acute high-output phase, so that regurgitant severity is graded under stable loading conditions. Optimization of guideline-directed heart-failure therapy, together with treatment of the underlying ventricular, pulmonary, and volume substrate, remains the first step, with severity re-graded once decongestion is complete. When regurgitation remains clinically significant despite optimal medical therapy, heart-team evaluation should determine candidacy for transcatheter mitral intervention, integrating symptom burden, ventricular function, anatomical suitability, and operative risk. Transcatheter mitral therapies—transcatheter edge-to-edge repair and, in selected anatomies, transcatheter mitral valve implantation—have broadened the options for patients at prohibitive surgical risk; however, patient selection, anatomical assessment, and several procedural and device-related issues remain incompletely resolved, highlighting the need for individualized, heart-team-based decision-making in this population [23].

### 3.2. Mechanisms and Acute, Potentially Reversible Hemodynamic Valvular Dysfunction

Four interacting mechanisms underpin cardiac dysfunction after COVID-19. First, direct endothelial and myocardial involvement: SARS-CoV-2 enters cells via ACE2, and autopsy and biopsy series have demonstrated viral presence within cardiac tissue together with endothelialitis and microvascular injury [1,2,20]. Valvular interstitial and endothelial cells express ACE2, providing a plausible substrate for inflammatory valvular involvement, although confirmed structural valvular infection remains rare and is documented mainly in isolated reports [11,17]. COVID-19-associated endotheliopathy—endothelial activation, glycocalyx disruption, and a prothrombotic, pro-inflammatory phenotype—is increasingly recognized as a unifying short- and long-term vascular substrate that links acute injury to persistent post-acute dysfunction [24]. At the same time, SARS-CoV-2-specific myocardial injury should be distinguished from the generic myocardial depression of sepsis and critical illness: sepsis-induced cardiomyopathy is a largely reversible, cytokine- and mitochondrial-driven dysfunction common to severe infection of any cause [25], so that some abnormalities observed in critically ill COVID-19 patients reflect shared critical-illness physiology rather than a virus-specific valvular or myocardial process.

Second, systemic hyperinflammation: the cytokine response—dominated by interleukin-6 (IL-6), tumor necrosis factor-α, and IL-1β—drives myocardial edema, depresses contractility, and promotes a procoagulant, pro-inflammatory endothelial phenotype that is mechanistically central to HFpEF [8,9]. In an Eastern European COVID-19 sepsis cohort, IL-6 together with cardiac biomarkers independently predicted adverse outcomes, and exploratory analysis suggested a modulating role for tocilizumab [15]. Inflammation is best regarded as an upstream contributor rather than a direct cause of valvular regurgitation. The proximate mechanisms that generate functional mitral and tricuspid regurgitation are ventricular remodeling, mitral and tricuspid annular dilatation, altered loading conditions, pulmonary hypertension, and right ventricular dysfunction; the inflammatory and endothelial milieu acts on these intermediaries rather than on the valve leaflets themselves.

Third, micro- and macrovascular thrombosis: COVID-19 is a profoundly prothrombotic state. In a Romanian cohort of 395 hospitalized patients, pulmonary embolism occurred in 11.9% and was associated with markedly elevated D-dimer, fibrinogen, and PT/INR, ICU transfer in roughly 60%, and 25.5% in-hospital mortality [13]. Acute right ventricular pressure overload from pulmonary embolism is a recognized contributor to functional tricuspid regurgitation and right-heart failure.

**Figure 1:**
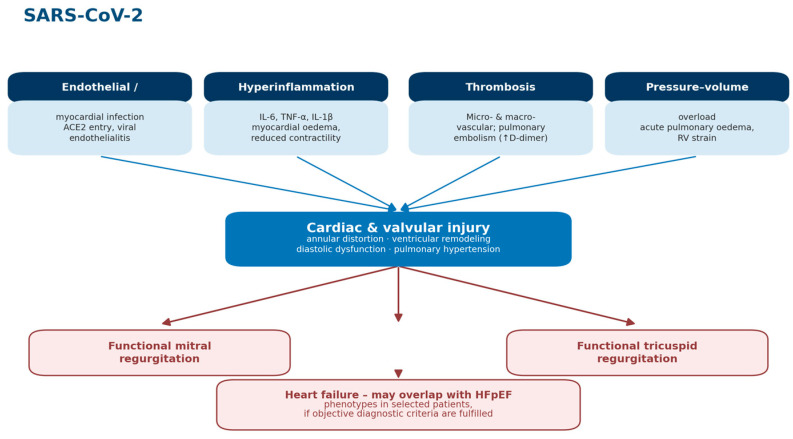
Convergent mechanistic pathways linking SARS-CoV-2 infection to valvular and ventricular dysfunction. Four interacting mechanisms—direct endothelial/myocardial infection via angiotensin-converting enzyme 2 (ACE2); systemic hyperinflammation (interleukin-6 [IL-6], tumor necrosis factor-α [TNF-α], IL-1β); micro- and macrovascular thrombosis including pulmonary embolism; and pressure–volume overload from acute pulmonary edema and right ventricular (RV) strain—converge on cardiac and valvular injury. The resulting annular distortion, ventricular remodeling, diastolic dysfunction, and pulmonary hypertension contribute to functional mitral and tricuspid regurgitation and heart failure with preserved ejection fraction (HFpEF). Blue arrows indicate the convergence of upstream SARS-CoV-2-related pathophysiological mechanisms toward cardiac and valvular injury. Red arrows indicate the proposed downstream clinical manifestations, including functional mitral regurgitation, functional tricuspid regurgitation, and heart-failure phenotypes. The arrows represent conceptual mechanistic associations and do not necessarily imply a direct causal or strictly sequential relationship.

Fourth, pressure–volume overload and cardio-inflammatory phenotypes: acute pulmonary edema (APE) reflects the intersection of inflammatory, endothelial, and cardiac injury. In a Romanian cohort, APE occurred in 36.2% of hospitalized patients and carried a four-fold increase in mortality (43.5% vs. 12.3%); elevated NT-proBNP, troponin I, and IL-6 independently predicted both APE and death, and at three months 39% of survivors showed fibrotic CT changes and 37% had restrictive ventilatory defects [14]. This sustained loading and remodeling provides the hemodynamic milieu for secondary valvular regurgitation and HFpEF. The convergence of these four mechanistic pathways and their downstream effects on ventricular and valvular function is summarized in Figure 1.

### 3.3. Spectrum of Valvular Involvement and Persistent Post-Acute Abnormalities

**Figure 2:**
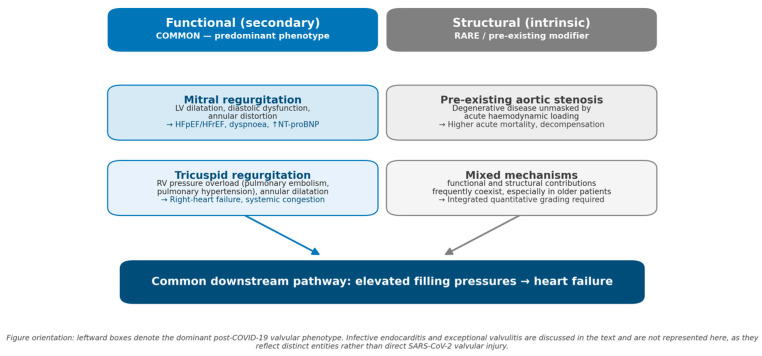
Spectrum of valvular involvement in the post-COVID-19 setting, classified as functional (secondary) versus structural (intrinsic). Functional regurgitation—predominantly mitral and tricuspid—appears to be the more common phenotype, arising from left ventricular (LV) dilatation and diastolic dysfunction and from right ventricular (RV) pressure overload, respectively. Structural disease is rare and largely confined to pre-existing degenerative aortic stenosis unmasked by acute loading and to isolated reports of valvular inflammation/endocarditis. Both pathways converge on elevated cardiac filling pressures and heart failure. HFpEF, heart failure with preserved ejection fraction; HFrEF, heart failure with reduced ejection fraction; NT-proBNP, N-terminal pro–B-type natriuretic peptide. To minimize overlap with Table 2, this schematic is intended to convey the functional-versus-structural dichotomy at a mechanistic level rather than to restate the individual findings tabulated there; the directness and strength of the evidence underpinning each category are classified separately in Table 1. Blue and gray arrows indicate the conceptual convergence of functional and structural valvular mechanisms, respectively, toward elevated cardiac filling pressures and heart failure. The arrows represent clinically plausible associations rather than direct or exclusively causal pathways.

Available evidence suggests that clinically relevant post-COVID valvular abnormalities are more often secondary/functional than primary structural lesions, although dedicated prospective valvular studies remain scarce. Secondary mitral regurgitation arises from left ventricular dilatation and diastolic dysfunction, while secondary tricuspid regurgitation follows right ventricular and annular dilatation driven by pulmonary hypertension and embolic pressure overload [11,12,26]. Reports of new structural valve disease attributable directly to SARS-CoV-2 are scarce; isolated cases of valvular inflammation and culture-negative endocarditis have been described but do not constitute a population-level signal [11,17]. Infective endocarditis reported after COVID-19 is most often related to healthcare exposure, indwelling catheters, or immune dysregulation and should not be interpreted as evidence of direct SARS-CoV-2 valvulitis; the two entities are mechanistically distinct and warrant clear separation. Pre-existing degenerative valvular disease, conversely, is a powerful modifier of acute COVID-19 outcome: an international registry of patients with severe VHD reported 30-day mortality approaching 50%, and a multicenter cohort found that pre-existing cardiovascular disease, including severe valvular disease, independently increased in-hospital mortality and myocardial injury [10,21]. The spectrum of post-COVID-19 valvular involvement, classified according to functional versus structural mechanisms, is illustrated in Figure 2.

**Table 2:** Predominant valvular findings reported in association with COVID-19 and their proposed mechanisms.

Valvular Finding	Type	Dominant Mechanism	Clinical Correlate
Mitral regurgitation	Functional	LV dilatation, diastolic dysfunction, annular distortion	HFpEF/HFrEF, dyspnea, elevated NT-proBNP
Tricuspid regurgitation	Functional	RV pressure overload (PE, pulmonary hypertension), annular dilatation	Right-heart failure, congestion
Aortic stenosis (pre-existing)	Structural/modifier	Degenerative; unmasked by acute loading	Higher acute mortality, decompensation
Infective endocarditis after COVID-19 (distinct from valvulitis)	Structural (rare)	Secondary bacterial/fungal infection (healthcare exposure, catheters, host vulnerability); not direct SARS-CoV-2 valvular infection	Rare; uncertain causal attribution to SARS-CoV-2

### 3.4. Incident Heart Failure After Recovery

Across cohorts, biomarker-defined cardiac stress is the most consistent predictor of adverse outcome. NT-proBNP and troponin elevation independently forecast mortality, ICU admission, and heart-failure phenotypes [14,15,27]. Heart-failure incidence rises in the post-acute period, with elevated 12-month hazards for incident HF among survivors [5]. The persistence of fibrotic changes on lung computed tomography and of restrictive ventilatory defects at three months represents pulmonary, rather than cardiac or valvular, remodeling; nonetheless, it identifies a subset of survivors who warrant continued cardiopulmonary follow-up [14]. Among hospitalized patients, HFpEF is disproportionately represented, reflecting the shared cardiometabolic and inflammatory substrate, and COVID-19 may precipitate overt decompensation in patients with previously subclinical diastolic dysfunction [9]. A diagnosis of HFpEF in this context should not be inferred from dyspnea, elevated natriuretic peptides, or preserved ejection fraction alone; objective evidence of elevated left ventricular filling pressures, together with corroborating structural or functional abnormalities, is required. Hospital-acquired secondary infection during admission may further compound cardiac outcomes [16].

Several design features of the studies summarized in Table 3 limit direct inference about valvular outcomes and should be read alongside it. Almost none included pre-infection (baseline) echocardiography, and serial standardized echocardiography with quantitative valve grading (vena contracta, effective regurgitant orifice area, regurgitant volume) was rarely performed; valvular regurgitation was therefore seldom an endpoint specified a priori. Most studies were designed to capture myocardial injury, ventricular dysfunction, pulmonary embolism, pulmonary hypertension, or general echocardiographic abnormalities rather than incident or worsening regurgitation, and several lacked pre-infection imaging entirely. The included evidence also spans different phases of the pandemic, from the pre-vaccination period through later vaccinated and variant-dominant eras, which further limits comparability. Taken together, these features reinforce that statements about a predominant post-COVID functional valvular phenotype are hypothesis-generating rather than confirmatory.

**Figure 3:**
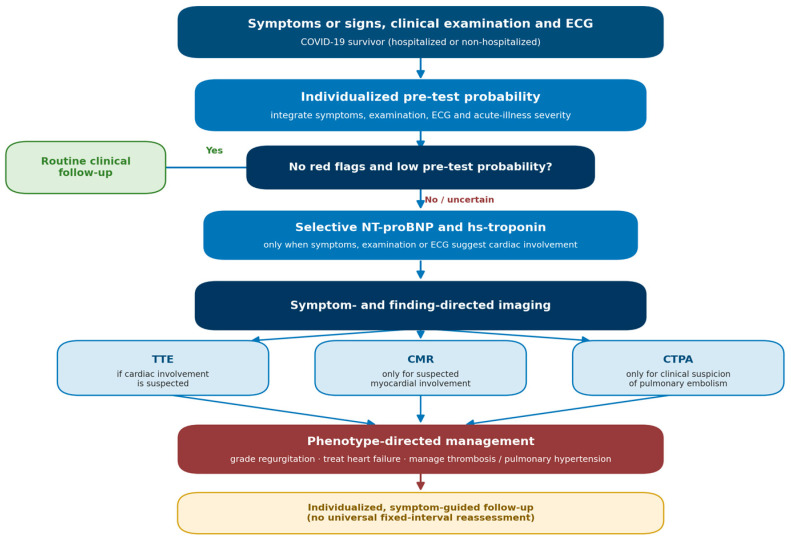
Proposed symptom- and risk-directed, multimodality follow-up pathway for cardiovascular surveillance of COVID-19 survivors. Evaluation begins with symptom assessment, clinical examination, and electrocardiography, from which an individualized pre-test probability of cardiac involvement is derived. Cardiac biomarkers (NT-proBNP and high-sensitivity troponin) and targeted imaging are obtained selectively in survivors with suggestive symptoms or signs, or an elevated pre-test probability, rather than as universal screening. Transthoracic echocardiography (TTE) with global longitudinal strain is the first-line imaging test; cardiac magnetic resonance (CMR) is reserved for suspected myocardial injury; and computed tomography pulmonary angiography (CTPA) is performed only when pulmonary embolism (PE) is clinically suspected. Routine interleukin-6 measurement and biomarker-triggered CTPA are not recommended. Phenotype-guided management and individualized reassessment then follow, with remote monitoring and telemedicine—including cardiac device telemetry and wearable, multiparametric platforms—incorporated for survivors with heart failure, arrhythmias, or cardiac implantable electronic devices. The algorithm is an expert-pragmatic proposal rather than a validated, evidence-based guideline, and requires prospective validation before routine clinical adoption. It represents an unvalidated, expert-opinion framework and is intended to complement, not replace, established consensus guidance, which favors symptom- and risk-directed rather than universal evaluation [28,29]. HF, heart failure. ECG, electrocardiography; HF, heart failure.

On the basis of the available evidence and its limitations, a pragmatic symptom- and risk-directed multimodality follow-up pathway for cardiovascular surveillance after COVID-19 is proposed in Figure 3.

### 3.5. Diagnostic and Multimodality Imaging Approach

Transthoracic echocardiography remains the first-line modality, enabling quantification of regurgitant severity, chamber dimensions, ventricular function, and estimated pulmonary artery pressures, and should follow standardized recommendations for grading native valvular regurgitation [30]. In a large international echocardiographic survey, abnormalities were frequent in hospitalized COVID-19 patients, with left and right ventricular dysfunction predominating and a minority showing severe disease [12]. Global longitudinal strain detects subclinical myocardial dysfunction not evident on ejection fraction alone and has revealed persistent abnormalities in post-COVID survivors [31,32]. Cardiac magnetic resonance characterizes myocardial edema, fibrosis, and late gadolinium enhancement; an early prospective study reported abnormal findings in a high proportion of recently recovered patients, and subsequent troponin-stratified imaging confirmed residual injury in a meaningful subset [18,33]. Computed tomography pulmonary angiography confirms embolic burden, the principal driver of acute right-heart strain [13]. Biomarkers—NT-proBNP, high-sensitivity troponin, and IL-6—complement imaging by stratifying risk and signaling cardio-inflammatory decompensation [14,15,27]. Early cardiac magnetic resonance reports of frequent abnormalities should, however, be interpreted alongside more recent controlled and longitudinal data. In the population-based Rotterdam Study, which uniquely incorporated knowledge of pre-COVID cardiovascular status, most non-hospitalized participants had normal ventricular function and tissue-characterization parameters, with only small longitudinal changes in left ventricular ejection fraction and global longitudinal strain [19]. The apparent burden of post-COVID cardiac involvement therefore varies substantially with acute-disease severity, hospitalization status, vaccination, reinfection, and viral era. A contemporary meta-analysis of long-COVID cardiovascular outcomes reported effect sizes more modest than early pandemic estimates [34], and a large vaccination-era cohort found that the excess cardiovascular risk after infection was markedly attenuated in vaccinated individuals and concentrated in the first weeks after acute illness [35].

### 3.6. Differentiating Incidental Degenerative VHD from Post-COVID Functional Regurgitation

A central clinical challenge—particularly in older adults—is distinguishing valvular abnormalities that pre-dated infection from those that are genuinely secondary to COVID-19. This distinction matters because management diverges: degenerative structural disease may warrant intervention per current valvular guidelines, whereas functional regurgitation is generally treated by addressing the underlying ventricular, pulmonary, and volume substrate [22]. Several features help orient the assessment. Functional regurgitation typically shows structurally normal leaflets with a dilated annulus and ventricle, central regurgitant jets, severity that tracks with loading conditions and pulmonary pressures, and improvement as inflammation, volume overload, and pulmonary hypertension resolve. Degenerative disease, by contrast, shows intrinsic leaflet abnormality (calcification, thickening, prolapse, restricted excursion), more fixed severity, and a chronic pattern of remodeling that does not reverse with decongestion. These features are orienting rather than absolute. Secondary regurgitation is not invariably central—eccentric jets occur with asymmetric leaflet tethering—and primary degenerative regurgitation may occasionally produce a central jet; mixed mechanisms are common, especially in older patients. Severity should therefore not be inferred from jet direction alone but from an integrated, quantitative assessment incorporating vena contracta width, effective regurgitant orifice area, regurgitant volume, pulmonary-vein flow, chamber dimensions, annular geometry, right ventricular function, and prevailing loading conditions. For tricuspid regurgitation, a contemporary multiparametric grading scheme should be applied, and the contributions of mechanical ventilation, pulmonary vascular disease, and atrial functional tricuspid regurgitation should be weighed explicitly.

Practically, comparison with any prior echocardiogram is the single most useful step; where unavailable, the combination of leaflet morphology, jet characteristics, chamber geometry, and the trajectory of regurgitant severity over serial studies after clinical stabilization provides the strongest discrimination. Because COVID-19 may also unmask previously subclinical degenerative disease or subclinical diastolic dysfunction—rather than create de novo structural lesions—the most defensible interpretation in many elderly patients is that infection acts as a precipitant or amplifier rather than the sole cause [21,36]. Reassessment after recovery, rather than during the acute inflammatory and high-output phase, reduces the risk of overcalling functional regurgitation as fixed structural disease. A practical comparison of the principal echocardiographic, hemodynamic, and temporal features distinguishing post-COVID functional regurgitation from incidental degenerative VHD is provided in Table 4.

### 3.7. Clinical Implications for Cardiologists and Internists

From a practical standpoint, the reviewed evidence supports a lower threshold for cardiovascular evaluation in COVID-19 survivors who present with specific features. Echocardiographic assessment is reasonable when a survivor has persistent or unexplained dyspnea, disproportionate fatigue, elevated or rising NT-proBNP or troponin, documented pulmonary embolism, new peripheral edema, a newly detected murmur, or a known history of valvular or other structural heart disease [22,30]. In such patients, transthoracic echocardiography with attention to valvular function, chamber geometry, diastolic indices, and estimated pulmonary pressures provides the most information for least cost.

For internists managing these patients outside specialized cardiology units, a pragmatic sequence is to (i) measure natriuretic peptides and troponin when cardiac symptoms are present; (ii) arrange echocardiography when biomarkers are elevated or symptoms persist; (iii) treat heart failure and pulmonary embolism according to current guidelines, including guideline-directed medical therapy for HF and appropriate anticoagulation for confirmed thromboembolism [22,36,37,38]; and (iv) refer for specialist valvular assessment when structural disease is suspected or when functional regurgitation remains significant after decongestion and resolution of the acute illness. Importantly, decisions about valvular intervention should generally be deferred until after recovery, when regurgitant severity can be reassessed under stable loading conditions, unless the clinical situation is critical [10,22].

This follow-up strategy must also account for how surveillance can realistically be delivered outside the hospital, a requirement made explicit by the pandemic itself. Many survivors with pre-existing valvular disease, secondary mitral or tricuspid regurgitation, heart failure, arrhythmias, or cardiac implantable electronic devices (CIEDs) required continuity of care at a time when in-person access had to be minimized. Remote monitoring and telemedicine were central to this task: converting routine in-hospital device checks to home monitoring allowed early detection of arrhythmic events, heart-failure alerts, and device-related findings while limiting hospital exposure, and was associated with fewer admissions for critical events, including heart failure, during lockdown periods [39]. In the post-COVID era, these systems retain a clear role in the longitudinal management of survivors with heart failure, arrhythmias, and CIEDs, and can be embedded in the biomarker-guided pathway proposed here as a means of detecting clinical deterioration between scheduled assessments.

Wearable and multiparametric monitoring deserve specific consideration in survivors with transient or potentially reversible ventricular dysfunction—for example, after COVID-19–associated myocardial injury, a myocarditis-like presentation, acute decompensated heart failure, or valvular-related heart failure—because the trajectory of recovery is often uncertain during early reassessment. In this vulnerable phase, the wearable cardioverter-defibrillator can provide temporary protection against sudden cardiac death while simultaneously serving as a multiparametric monitoring platform; its structured use, combined with serial reassessment of ventricular function, may help avoid both unnecessary hospitalizations and premature, potentially avoidable permanent defibrillator implantation [40]. This approach aligns post-COVID follow-up with the broader principle of deferring irreversible device decisions until ventricular recovery has been adequately characterized.

In practical terms, these considerations support a simple triage. Survivors with persistent or unexplained cardiopulmonary symptoms, rising natriuretic peptides or troponin, a newly detected murmur, documented pulmonary embolism, or known structural heart disease should undergo transthoracic echocardiography; survivors with heart failure, significant arrhythmia, or a CIED are candidates for remote monitoring and, where appropriate, wearable multiparametric surveillance during the reassessment window; patients in whom structural disease is confirmed, or in whom functional regurgitation remains significant after decongestion and recovery, should be referred to a heart-valve team; and asymptomatic survivors with normal biomarkers and no high-risk features can reasonably continue routine clinical follow-up. This tiered approach concentrates resources on the patients most likely to benefit while avoiding over-investigation of those at low risk.

## 4. Discussion

This review synthesizes a coherent, if necessarily cautious, picture: the same endothelial, thrombotic, and hemodynamic mechanisms that predispose survivors to heart failure—which may overlap with HFpEF phenotypes in selected patients when objective diagnostic criteria are fulfilled—also underlie the secondary, functional regurgitation that dominates the post-COVID valvular phenotype. Rather than a discrete new valvulopathy, SARS-CoV-2 most plausibly acts as a precipitant or amplifier of secondary regurgitation and ventricular remodeling, with cardiac biomarkers serving as the unifying thread for risk stratification. Because direct prospective valvular data remain limited, these conclusions are framed as the most defensible synthesis of indirect and mechanistic evidence rather than as established fact.

### 4.1. Comparison with Other Reviews

Prior syntheses of post-COVID cardiovascular disease have focused chiefly on myocarditis, arrhythmia, myocardial injury, and incident heart failure, with valvular disease typically relegated to a brief mention [6,7,41]. Reviews of long-COVID cardiac sequelae emphasize myocardial fibrosis and autonomic dysfunction but rarely integrate the valvular consequences of right-heart pressure overload [6,32]. Reviews dedicated to COVID-19 and HFpEF have clarified the inflammatory–endothelial substrate but do not systematically address the valvular interface [9]. Our synthesis differs in three respects. First, it foregrounds functional valvular pathology as a frequently overlooked and, on currently available evidence, probably predominant valvular phenotype, explicitly linking it to pulmonary embolism and acute pulmonary edema rather than to direct valvulitis. Second, it provides an explicit framework for differentiating incidental degenerative VHD from post-COVID functional regurgitation, a recurrent bedside problem absent from most prior reviews. Third, it integrates granular regional cohort data quantifying embolic burden, the cardio-inflammatory APE phenotype, and biomarker-driven sepsis outcomes [13,14,15].

### 4.2. Strengths

The principal strength of this review is its mechanistic integration: it connects endothelial, thrombotic, inflammatory, and hemodynamic pathways into a single explanatory framework for both valvular dysfunction and heart failure. A second strength is the incorporation of well-characterized regional cohort evidence, lending quantitative texture—prevalence, biomarker thresholds, and follow-up imaging—to mechanisms often discussed only qualitatively. Third, the review carefully separates functional from structural valvular disease and provides practical, guideline-anchored guidance for cardiologists and internists, including an explicit differential-assessment framework and a biomarker-guided follow-up algorithm. Fourth, contemporary guideline references, including the 2025 ESC/EACTS valvular guidelines, anchor the recommendations to current standards [22].

### 4.3. Limitations

Several limitations temper these conclusions. As a narrative review, this work did not apply systematic search protocols, PROSPERO registration, or quantitative meta-analysis, and is therefore susceptible to selection and interpretation bias. The literature on post-COVID valvular disease specifically is sparse and heterogeneous: few studies were designed to capture valvular endpoints, definitions of valvular severity and imaging protocols vary, and the true incidence of post-COVID functional regurgitation is likely under-ascertained. The supporting regional cohorts are retrospective, single-center, and observational, limiting causal inference and generalizability. Confounding by pre-existing cardiovascular disease is pervasive. Disentangling direct viral effects from the consequences of critical illness, mechanical ventilation, and prolonged immobilization remains difficult. Much of the evidence predates current variants and contemporary vaccination coverage, which may attenuate the cardiac phenotype in more recent populations. For these reasons, the central claims are deliberately framed as probabilistic syntheses requiring prospective confirmation. Therefore, the findings should be interpreted as a mechanistic and clinically oriented synthesis rather than as pooled evidence.

### 4.4. Strength and Classification of the Available Evidence

A recurring difficulty in this field is that statements about post-COVID valvular dysfunction rest on evidence of differing directness and strength. To make this explicit, Table 1 classifies the evidence underlying the present synthesis according to the directness of its bearing on valvular outcomes: directly documented new or worsening valvular dysfunction; transient functional regurgitation during acute critical illness; persistent post-acute valve-specific abnormalities; plausible mechanistic links inferred from ventricular or pulmonary disease; and pre-existing VHD acting as a prognostic modifier—together with the inferences each can reasonably support and its principal limitations. Directly documented de novo valvular disease remains the sparsest category, whereas the prognostic role of pre-existing severe VHD and the mechanistic plausibility of secondary functional regurgitation are comparatively better supported. Conclusions about de novo structural valve disease should therefore be read as hypothesis-generating, while the proposition that post-COVID valvular abnormalities are predominantly secondary and functional is supported indirectly but consistently. Readers are encouraged to weight the clinical suggestions in this review accordingly.

### 4.5. Future Directions

Prospective, multicenter longitudinal studies with protocolized echocardiography, strain imaging, and cardiac magnetic resonance are needed to define the true trajectory and reversibility of post-COVID valvular dysfunction, ideally with serial assessment that separates the acute high-output phase from the recovered steady state. Standardized valvular endpoints should be embedded in long-COVID registries. Biomarker-guided follow-up algorithms—anchored on NT-proBNP, troponin, and IL-6—warrant prospective validation as triage tools, and the cardiac effects of anti-inflammatory and anticoagulant strategies, as well as guideline-directed HF therapies in this population, deserve dedicated evaluation.

## 5. Conclusions

Valvular dysfunction in the post-COVID-19 era appears predominantly functional, inflammation- and thrombosis-related, and tightly interwoven with the pathophysiology of heart failure, which may overlap with preserved-ejection-fraction phenotypes in selected patients when objective diagnostic criteria are fulfilled. Direct structural valve disease attributable to SARS-CoV-2 is rare; the more common and clinically important phenomenon appears to be secondary mitral and tricuspid regurgitation arising from right-heart pressure overload, diastolic dysfunction, and post-acute remodeling, while pre-existing severe valvular disease markedly worsens acute prognosis. Differentiating incidental degenerative VHD from genuinely post-COVID functional regurgitation—best done after recovery and with reference to prior imaging—is a practical priority for clinicians. Cardiac biomarkers, particularly NT-proBNP, troponin, and IL-6, are the most reliable predictors of decompensation and should inform a symptom- and risk-directed, multimodality follow-up strategy in high-risk survivors. Because dedicated longitudinal evidence on valvular outcomes specifically remains very limited, these conclusions are best regarded as a hypothesis-generating synthesis of largely indirect evidence, and prospective longitudinal studies with standardized valvular endpoints remain the principal research priority.

## Figures and Tables

**Table 1:** Classification of the evidence underpinning this review, by directness and strength. CMR, cardiac magnetic resonance; HF, heart failure; VHD, valvular heart disease.

Evidence Category	Representative Basis in This Review	Principal Inferences Supported	Strength and Interpretation
Directly documented new or worsening valvular dysfunction	Exceptional case reports of valvulitis and culture-negative endocarditis [11,17]	De novo structural valve disease attributable to SARS-CoV-2 is rare and of uncertain causation	Sparse; hypothesis-generating only
Transient functional regurgitation during acute critical illness	Acute echocardiographic findings during severe COVID-19, PE, pulmonary hypertension and RV overload [12,13]	Secondary MR/TR can arise acutely from loading and RV dysfunction and may be reversible	Moderate but largely load-dependent; rarely assessed with serial quantitative grading
Persistent post-acute valve-specific abnormalities	Longitudinal echocardiography and CMR after recovery, including pre-COVID-controlled data (Rotterdam) [18,19]	Most survivors show only small longitudinal changes; durable valve-specific abnormality is not well established	Limited and heterogeneous; few studies have pre-infection imaging
Plausible mechanistic links inferred from ventricular or pulmonary disease	Endothelial, thrombotic and inflammatory pathways; autopsy, PE and pulmonary-edema data [1,2,8,9,13,14,20]	Biological plausibility linking SARS-CoV-2 to functional regurgitation and HF phenotypes	Strong mechanistic plausibility; not direct proof of valvular causation
Pre-existing VHD acting as a prognostic modifier	Severe-VHD registry and cohort mortality data [10,21]	Pre-existing severe VHD markedly worsens acute COVID-19 prognosis	Consistent as a prognostic modifier; not evidence of de novo valvular injury

**Table 3:** Key studies evaluating cardiovascular, heart failure, echocardiographic, and valvular outcomes after COVID-19. Study-level design features relevant to valvular inference—baseline imaging, serial echocardiography, quantitative valve grading, vaccination/variant era, and a priori valvular endpoints—are summarized in the text immediately following the table.

Study	Population and Pandemic Era	Pre-Infection/Baseline Imaging	Serial Cardiac Imaging	Quantitative Valve Grading	Valvular Endpoint Specified a Priori	Main Finding	Relevance/Limitation
Xie et al., 2022 [5]	153,760 US veterans; early pandemic (pre-Omicron)	No	No	No	No	Increased incident HF, arrhythmia, thrombosis at 12 mo	Large but administrative claims data; no valvular endpoint, indirect
Puntmann et al., 2020 [18]	100 recovered; wild-type era	No	No	No	No	Frequent residual myocardial inflammation on CMR	Statistical corrections issued; single-center; no valve-specific endpoint
Dweck et al., 2020 [12]	1216 patients; first wave, acute	No	No	No	No	55% abnormal echocardiograms; LV/RV dysfunction	Severity-driven sampling bias; cross-sectional
Severe VHD Registry, 2021 [10]	136 severe pre-existing VHD; first wave	Yes (pre-existing VHD known)	No	Pre-existing grading only	No (prognostic modifier)	30-day mortality ~50%	Pre-existing severe VHD as prognostic modifier; no control group
Petersen et al. (Rotterdam Study), 2024	Population-based; pre- and post-COVID imaging available	Yes (pre-COVID CMR)	Yes (longitudinal)	No dedicated valve grading	No	Mostly normal ventricular function; only small longitudinal LVEF/GLS changes	Key contemporary control for pre-COVID status; argues against major persistent injury
Long-COVID CV meta-analysis, 2025	Pooled multi-cohort; mixed eras	Variable	Variable	No	No	Effect sizes more modest than early pandemic estimates	Heterogeneous; highlights attenuation with later eras
OpenSAFELY vaccination-era analysis, 2024	Large national cohort; vaccination era	No	No	No	No	Lower post-COVID cardiovascular risk in vaccinated, later-variant periods	Demonstrates era/vaccination heterogeneity; no valvular endpoint
Mateescu et al., 2025 [13]	395 hospitalized (RO); pre-Omicron	No	No	No	No	PE 11.9%; in-hospital mortality 25.5%; RV overload	No dedicated valvular endpoint; indirect mechanistic relevance only
Mateescu et al., 2025 [14]	127 hospitalized (RO); 3-mo follow-up	No	No	No	No	APE 36.2%; ~4-fold mortality	Pulmonary fibrotic CT changes and restrictive ventilatory defects; no serial valvular assessment or valve-specific endpoint
Mateescu et al., 2025 [15]	COVID-19 sepsis cohort (RO)	No	No	No	No	IL-6 and cardiac markers predict outcome	No dedicated valvular endpoint; exploratory tocilizumab analysis

**Table 4:** Features distinguishing post-COVID functional regurgitation from incidental degenerative valvular heart disease (VHD).

Feature	Post-COVID Functional Regurgitation	Incidental Degenerative VHD
Leaflet morphology	Structurally normal	Calcified, thickened, prolapsing or restricted
Regurgitant jet	Often central, but may be eccentric with asymmetric tethering	Often eccentric or lesion-directed, but may be central
Severity over time	Load-dependent; often improves with decongestion	Relatively fixed; progresses chronically
Chamber geometry	Dilated ventricle/annulus, pulmonary hypertension	Lesion-specific remodeling
Prior echocardiogram	Usually normal or mild	Pre-existing abnormality documented
Mixed mechanisms	Common, particularly in older patients; functional and structural contributions coexist	Requires integrated quantitative grading rather than jet direction alone

## Data Availability

No new data were generated or analyzed in this study. All information is derived from previously published studies, which are cited within the manuscript.

## Abbreviations

The following abbreviations are used in this manuscript:

ACE2	Angiotensin-converting enzyme 2
APE	Acute pulmonary edema
CMR	Cardiac magnetic resonance
COVID-19	Coronavirus disease 2019
CT	Computed tomography
CTPA	Computed tomography pulmonary angiography
HF	Heart failure
HFpEF	Heart failure with preserved ejection fraction
HFrEF	Heart failure with reduced ejection fraction
ICU	Intensive care unit
IL-1β	Interleukin-1 beta
IL-6	Interleukin-6
LV	Left ventricle/left ventricular
NT-proBNP	N-terminal pro–B-type natriuretic peptide
PASC	Post-acute sequelae of SARS-CoV-2 infection
PE	Pulmonary embolism
PT/INR	Prothrombin time/international normalized ratio
RV	Right ventricle/right ventricular
SARS-CoV-2	Severe acute respiratory syndrome coronavirus 2
TNF-α	Tumor necrosis factor alpha
TR	Tricuspid regurgitation
TTE	Transthoracic echocardiography
VHD	Valvular heart disease
